# *Nigella sativa* as an anti-inflammatory agent in asthma

**DOI:** 10.1186/s13104-018-3858-8

**Published:** 2018-10-19

**Authors:** Mukhtar Ikhsan, Nurul Hiedayati, Kazutaka Maeyama, Fariz Nurwidya

**Affiliations:** 1 Department of Pulmonology and Respiratory Medicine, Faculty of Medicine Universitas Indonesia, Persahabatan Hospital, Jalan Persahabatan Raya No.1, Rawamangun, Jakarta, 13230 Indonesia; 2 Department of Pulmonology and Respiratory Medicine, Faculty of Medicine and Health Sciences Universitas Islam Negeri Syarif Hidayatullah, Jakarta, Indonesia; 3 Department of Pharmacology, Faculty of Medicine and Health Sciences Universitas Islam Negeri Syarif Hidayatullah, Jakarta, Indonesia; 4 Department of Pharmacology, Informational Biomedicine, Ehime University Graduate School of Medicine, Shitsukawa, Toon, Ehime 791-0295 Japan; 5 Department of Nutrition, Faculty of Medicine Universitas Indonesia, Dr. Cipto Mangunkusumo Hospital, Jakarta, Indonesia

**Keywords:** *Nigella sativa*, Anti-inflammatory, Asthma

## Abstract

**Objective:**

*Nigella sativa* (*N. sativa*) has several pharmacological actions which include antioxidant, antidiabetic, anticancer, antitussive, immunomodulator, analgesic, antimicrobial, anti-inflammatory, spasmolytic, and bronchodilator. The purpose of this study is to measure the effectivity of *N. sativa* ethanol extract as anti-inflammation on peritoneal Wistar rat mast cells. The laboratory experiment was used to investigate the effectivity of *N. sativa* as an anti-inflammatory on mast cells. Six groups of mast cells were stimulated by C 48/80 to release histamine. Group 1 were without *N. sativa*, while group 2, 3, 4, 5, and 6 were given *N. sativa* with concentrations of 0.1 mg/ml, 0.2 mg/ml, 0.3 mg/ml, 0.4 mg/ml and 0.5 mg/ml, respectively. Histamine concentration was measured by high-performance liquid chromatography-fluorometry.

**Result:**

The study showed that *N. sativa* ethanol extract effectively inhibit histamine release from peritoneal Wistar rat mast cells proportionally to its concentration. *N.* sativa is effective as an anti-inflammation on mast cells by inhibition of histamine release and has no toxic effect on mast cell. *N. sativa* could be considered as a potential therapy for asthma therapy and prevention.

## Introduction

The seeds of the annual flowering plant, *Nigella sativa* (*N. Sativa*), have been prized for their healing properties and was popularly used in traditional medicine systems such as Greece, Ayurveda and Siddha [1]. *N. sativa* is also mentioned in the Bible [2]. Furthermore, Imam Bukhari and Imam Muslim have narrated that Prophet Muḥammad (peace be upon him) said: “Hold on to the use of the *habbat al*-*sauda* (*N. sativa*) for indeed it has a remedy for every disease except death” [3]. *N. sativa* has a broad spectrum of pharmacological actions, including antioxidant, antidiabetic, anticancer, antitussive, immunomodulator, analgesic, antimicrobial, anti-inflammatory, spasmolytic, and bronchodilator [4].

Asthma is one of the major health problems in the world. There is a difference in the prevalence of asthma in different countries that varies from 1 to 18%. In Indonesia asthma is the top ten cause of morbidity and mortality, as illustrated by Household Health Survey (Survei Kesehatan Rumah Tangga, SKRT) data in various provinces in Indonesia [5].

Asthma is a chronic inflammatory disease of the airways. Various inflammatory cells involved primarily mast cells, eosinophils, T lymphocytes, macrophages, neutrophils, and epithelial cells. Environmental factors and other factors play a role in causing or triggering airway inflammation in asthma. Inflammation is present in various degrees of asthma in both intermittent and persistent asthma. Inflammation can be found in various forms of asthma such as allergic asthma, non-allergic asthma, work asthma and asthma triggered by aspirin [6].

Mast cells are the most important cells in the early stages of an asthma attack. Mast cells have high-affinity immunoglobulin-E (IgE) receptors. Cross-linking IgE receptors with factors in mast cells activates mast cells. There is degranulation of mast cells that release preformed mediators such as histamine and proteases as well as newly generated mediators such as prostaglandin D2 and leukotriene. Mast cells also release cytokines such as tumor necrosis factor-α (TNF-α), interleukin (IL)-3, IL-4, IL-5 and granulocyte–macrophage-colony-stimulating factor (GM-CSF) [6].

The previous study showed that *N. sativa* significantly inhibited mast cell degranulation and reduced mast cells population induced by *Tricholoma terreum* [7]. Furthermore, *N. sativa* seed extract also decreased intestinal mast cell numbers and plasma mouse mast cell protease-1 (MMCP-1) in the hypersensitive murine model [8]. In the form of oil, *N. sativa* significantly decreases airway hyperresponsiveness through the reduced number of leukocytes, macrophages, eosinophils, and levels of several asthma-related interleukins [9]. In addition, Khaldi et al. have demonstrated *N. sativa* oil administration had anti-inflammatory effects by reducing interleukin 4 (IL-4) and nitrite oxide (NO) production in rat asthma model [10]. In a single-blind randomized clinical trial, *N sativa* supplementation with inhaled maintenance therapy increase asthma control test (ACT) score, improve lung function and reduce IgE levels in partly controlled asthma subjects [11]. The current study was conducted to assess the toxicity of *N. sativa* ethanol extract on mast cell and effectivity of *N. sativa* ethanol extract in histamine release inhibition from peritoneal Wistar rat mast cells which received stimulation by C 48/80.

## Main text

### Materials and methods

Identification of *N. sativa* seeds was performed at the Center of Biopharmaceutical Studies, Bogor Agricultural University, Bogor, Indonesia. In vivo and in vitro experiments were performed at the Department of Pharmacology, Informational Biomedicine, Ehime University Graduate School of Medicine, Ehime, Japan.

#### Preparation of *N. sativa* ethanol extract

The dried *N. sativa* seed was crushed and extracted with a ratio of 1 g of sample with 10 ml of 50% ethanol solvent for 12 h three times. The extraction results were filtered with Whitman filter paper and saturated with a vacuum rotator at a temperature of 30 °C.

#### Purification of peritoneal Wistar rat mast cells

The Wistar rats were housed in a temperature of 25 °C room with a sufficient light and dark cycle and a relative humidity of 55%. Irradiated food and pure water and were provided ad libitum. All procedures performed on the animals were approved by the Institutional Review Board (IRB) of Faculty of Medicine Universitas Islam Syarif Hidayatullah, Jakarta, Indonesia (Ethical Approval No. Un.01/F10/KP.01.1/KE.SP/05.12.001/2011) in 2011. Wistar rats were sacrificed by ether anesthesia. Cells from the peritoneal cavity were recovered by washing the peritoneal cavity with phosphate-buffered saline (PBS) containing heparin. Purification of mast cells was performed with a Percoll density gradient. The mast cells were then calculated by staining with Toluidin blue pH 2.5 and viable mast cells were counted by trypan blue paint.

#### Preparation of *N. sativa* solution

*Nigella sativa* extract was dissolved in dimethyl sulfoxide (DMSO), and then dissolved in Pipe Siragainan (PIPES) buffer. The 0.4% DMSO solution did not stimulate the release of histamine by mast cells, and PIPES buffer was used for the control group. Compound (C) 48/80 solution with a concentration of 10 μg/ml was used to stimulate the release of histamine from mast cells as much as 85%.

#### Measurement of histamine level

Histamine was measured by high-performance liquid chromatography (HPLC)-fluorometry according to manufacturer protocols. After 30 min incubated with C 48/80, 96 well plates were centrifuged at 3000 rpm for 5 min and 50 μl, supernatant was taken and placed on a 1.5 ml plastic tube and added 250 μl of 3% perchloric acid containing 5 mM Na2-ethylene-diamine-tetra-acetic acid (EDTA), after addition of 30 μl 2 M KOH/1 M KH2PO4 and centrifuged at 10,000×*g* for 15 min at 4° C, 50 μl supernatant was injected in TSKgel column of SP-2SW cation exchanger (Tosoh, Tokyo). To measure the total content of histamine in mast cells, the remainder of the mast cell solution at the well was destroyed by sonication then 50 μl was used to measure histamine.

Histamine was diluted with 0.25 M potassium phosphate with a flow rate of 0.6 ml/min, then labeled with *o*-phthalaldehyde under alkaline conditions and detected with F1080 Fluorometer (Hitachi, Tokyo) on excitation and emission 360 and 450 nm, respectively.

#### Calculation of histamine release percentages

The percentage of net histamine release (%) was calculated by the following formula = (histamine content of stimulated mast cell supernatant − histamine content in unstimulated mast cell supernatant)/(total histamine content − histamine content in unstimulated mast cells) × 100. Meanwhile, the percentage of spontaneous histamine release (%) was obtained by the following formula = (histamine content in unstimulated mast cell supernatant)/(total histamine content) × 100. As for the percentage of inhibition of histamine release (%), this formula was used = (histamine release in supernatant of mast cells stimulated in the absence of *N. sativa* extract − histamine release in mast cell supernatant stimulated by the presence of *N. sativa* extract)/(histamine release in mast cell supernatant stimulated in the absence of *N. sativa* extract) × 100.

#### Data processing and statistical analysis

All data were described as the mean ± standard error of the mean (SEM). The data were statistically analyzed using analysis of variances (ANOVA) followed by the least significant difference (LSD). The significance limit was p < 0.05.

### Results

#### Toxicity of *Nigella sativa*

**Fig. 1:**
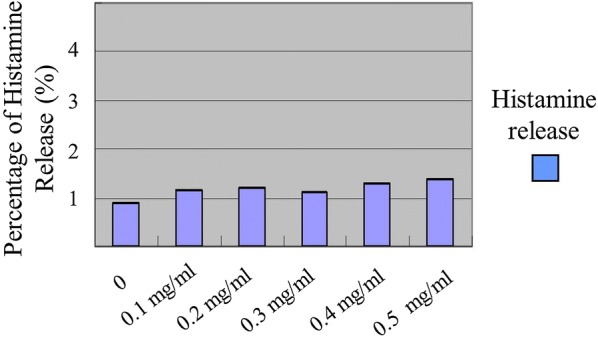
Effect of various concentrations of *N. sativa* ethanol extract on histamine release in mast cells without stimulation by C 48/80. Each data describes the mean ± SEM of 5 experiments with triplication (n: 15)

First, we examined the toxicity of *N. sativa* on mast cells. Figure 1 showed the histamine release from mast cells without stimulation by C 48/80. Without stimulation by C 48/80 compound, to a concentration of 0.5 mg/ml of *N. sativa* ethanol extract did not result in significant spontaneous histamine release (< 10%). One point 77% at the concentration of 0, 2.28% at the concentration of 0.1 mg/ml, 2.38% at the concentration of 0.2 mg/ml, 2.25% at the concentration of 0.3 mg/ml, 2.63% at the concentration of 0.4 mg/ml and 2.74% at the concentration of 0.5 mg/ml, respectively. The results did not differ significantly in the control group in which the histamine release is 1.77%. These data showed that *N. sativa* ethanol extract did not result in damage (lysis) to mast cells.

#### Effectivity of *Nigella sativa* in histamine release inhibition

**Fig. 2:**
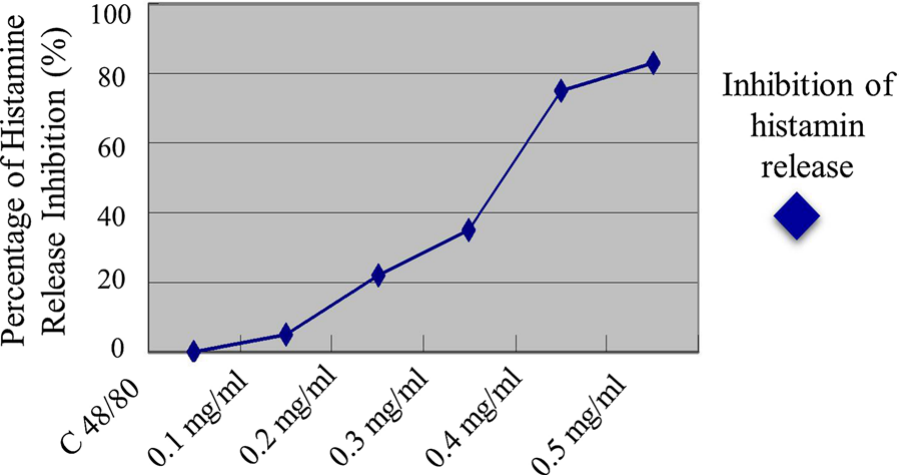
The effect of histamine release inhibition from mast cells stimulated by C 48/80. Each data describes mean ± SEM of 5 experiment with triplication (n: 15)

Next, we determined the effectivity of *N. sativa* ethanol extract in the histamine release inhibition. Figure 2 showed the effect of histamine release inhibition by *N. sativa* ethanol extract after stimulated by C 48/80. The greater concentration of *N. sativa* ethanol extract resulted the greater inhibition effect, 4.54% in concentration of 0.1 mg/ml, 22.4% in concentration of 0.2 mg/ml, 35.38% in the concentration of 0.3 mg/ml, 75.43% in the concentration of 0.4 mg/ml and 83.42% in the concentration of 0.5 mg/ml. The study showed that *N. sativa* ethanol extract can inhibit histamine release from peritoneal Wistar rat mast cells stimulated by C 48/80 proportionally to its concentration.

### Discussion

Treatment of asthma by an agent with sufficient safety level is important and under intensive investigation. With the known pharmacological action of *N. Sativa*, it is necessary to determine the toxicity aspect at the cellular level. We found that *N. Sativa* caused no toxicity to mast cells. This study results are similar to the study result by Dollah et al. who performed a study to determine the toxic effect of *N. sativa* powder on the kidney function which was evaluated by serum urea and creatinine and through histopathological examination of kidney tissue. They found that there is no toxic effect on kidney function of *N. sativa* at different doses for 5-week period of treatment [12]. Another study also showed that supplementation of *N. sativa* up to the dose of 1 g/kg supplemented for a period of 28 days resulted in no changes in liver enzymes level and did not cause any toxicity effect on the liver function [13].

Mast cells played important role in the inflammation of airway in asthma due to their capacity to release histamine. We showed in this study that *N. sativa* was effective in the inhibition of histamine release by mast cells. The previous study has revealed that thymoquinone (one of the active substances of *N. sativa*) attenuates the inflammatory response in activated mast cells by blocking transcription and production of TNFα through modulation of the proinflammatory transcription factor nuclear factor-κB (NF-κB) [14]. Furthermore, thymoquinone can inhibit leukotriene formation in human blood, through the inhibition of both 5-lipoxygenase and leukotriene C4 (LTC4)-synthase activity [15]. Thymoquinone has a function as an anti-inflammatory, anti-oxidant, and anti-allergic which highlights the potency of thymoquinone to treat airway hypersensitivity-related diseases including asthma [16].

Boskabady et al. studied the preventive effect of a hydro-ethanolic extract of *N. sativa* on the tracheal responsiveness and white blood cell count in the lung lavage fluid of sensitized guinea pigs. The results demonstrate the preventive effect of the *N. sativa* extract on the tracheal response and lung inflammation in sensitized guinea pigs [17].

Several clinical studies have been performed to assess the anti-inflammation features of *N. Sativa* in asthmatic subjects. Boskabady et al. studied the effects of *N. sativa* boiled extract as anti-asthma in asthmatic patients and the results showed that the extract caused significant increases in all measured pulmonary function tests [18]. The prophylactic effects of *N. sativa* boiled extract was also confirmed in a study involving 29 asthmatic patients. Among the *N. sativa* group, the use of oral inhalers and beta-agonists, oral corticosteroids, oral theophylline, and even corticosteroid inhalers decreased at the end of the study while no apparent change in the use of drugs in the control group [19].

In a study, treatment with *N. sativa* oil supplementation resulted in improvement on clinical and inflammatory parameters of asthma such as ACT score, pulmonary function test, blood eosinophils and total serum Immunoglobulin E [20]. Moreover, supplementation with *N. sativa* oil improves interferon-γ (IFN-γ)/IL-4 balance and ACT in children with asthma [21]. These studies suggest that *N. sativa* might improve the clinical outcome of a patient with asthma through the various pathways within the spectrum of asthma pathophysiology.

As a conclusion, *N. sativa* ethanol extract has no toxic effect on mast cells and is effective as an anti-inflammatory by histamine release inhibition from mast cells. The anti-inflammatory features of *N. sativa* are promising in the prevention and therapy of asthma.

## Limitations

The current study only investigated the effect of *N. sativa* on histamine release, meanwhile, various active effector substrate are involved in the asthma symptoms, including leukotriene. Furthermore, there is a need to explore what signaling pathway that is inhibited by *N. sativa*, considering to the complexity of interleukins that mediate the release of histamine by mast cells.

## Abbreviations

ACT: asthma control test
DMSO: dimethyl sulfoxide
EDTA: ethylene-diamine-tetra-acetic acid
GM-CSF: granulocyte–macrophage-colony-stimulating factor
IFN-γ: interferon-γ
IgE: immunoglobulin-E
IL: interleukin
LTC4: leukotriene C4
MMCP-1: mast cell protease-1
NO: nitrite oxide
NF-κB: nuclear factor-κB
PBS: phosphate-buffered saline
PIPES: Pipe Siragainan
SEM: standard error of the mean
SKRT: *survei kesehatan rumah tangga* (Household Health Survey)
TNF-α: tumor necrosis factor-α
